# Fed-batch process for the psychrotolerant marine bacterium *Pseudoalteromonas haloplanktis*

**DOI:** 10.1186/1475-2859-9-72

**Published:** 2010-09-21

**Authors:** Boris Wilmes, Angelika Hartung, Michael Lalk, Manuel Liebeke, Thomas Schweder, Peter Neubauer

**Affiliations:** 1 Institute of Marine Biotechnology, W.-Rathenau-Str. 49, D-17489 Greifswald, Germany; 2 Pharmaceutical Biotechnology, Institute of Pharmacy, Ernst-Moritz-Arndt-University, F.-L.-Jahn-Str. 17, D-17487 Greifswald, Germany; 3 Bioprocess Engineering Laboratory, University of Oulu, P.O.Box 4300, FI-90014 Oulu, Finland; 4 Department of Internal Medicine IV, University of Tübingen, Otfried-Müller Str.10, D-72076 Tübingen, Germany; 5 Competence Center - Functional Genomics, Institute of Pharmacy, Ernst-Moritz-Arndt-University, F.-L.-Jahn-Str. 17, D-17487 Greifswald, Germany; 6 Biomolecular Medicine, Department of Surgery and Cancer, Faculty of Medicine, Imperial College London, London, UK SW7 2AZ, UK; 7 Laboratory of Bioprocess Engineering, Department of Biotechnology, Technical University Berlin, Ackerstr. 71-76, D-13355 Berlin, Germany

## Abstract

**Background:**

*Pseudoalteromonas haloplanktis *is a cold-adapted γ-proteobacterium isolated from Antarctic sea ice. It is characterized by remarkably high growth rates at low temperatures. *P. haloplanktis *is one of the model organisms of cold-adapted bacteria and has been suggested as an alternative host for the soluble overproduction of heterologous proteins which tend to form inclusion bodies in established expression hosts. Despite the progress in establishing *P. haloplanktis *as an alternative expression host the cell densities obtained with this organism, which is unable to use glucose as a carbon source, are still low. Here we present the first fed-batch cultivation strategy for this auspicious alternative expression host.

**Results:**

The key for the fed-batch cultivation of *P. haloplanktis *was the replacement of peptone by casamino acids, which have a much higher solubility and allow a better growth control. In contrast to the peptone medium, on which *P. haloplanktis *showed different growth phases, on a casamino acids-containing, phosphate-buffered medium *P. haloplanktis *grew exponentially with a constant growth rate until the stationary phase. A fed-batch process was established by feeding of casamino acids with a constant rate resulting in a cell dry weight of about 11 g l^-1 ^(OD_540 _= 28) which is a twofold increase of the highest densities which have been obtained with *P. haloplanktis *so far and an eightfold increase of the density obtained in standard shake flask cultures.

The cell density was limited in the fed-batch cultivation by the relatively low solubility of casamino acids (about 100 g l^-1^), which was proven by pulse addition of casamino acid powder which increased the cell density to about 20 g l^-1 ^(OD_540 _= 55).

**Conclusion:**

The growth of *P. haloplanktis *to higher cell densities on complex medium is possible. A first fed-batch fermentation strategy could be established which is feasible to be used in lab-scale or for industrial purposes. The substrate concentration of the feeding solution was found to influence the maximal biomass yield considerably. The bottleneck for growing *P. haloplanktis *to high cell densities still remains the availability of a highly concentrated substrate and the reduction of the substrate complexity. However, our results indicate glutamic acid as a major carbon source, which provides a good basis for further improvement of the fed-batch process.

## Background

*Pseudoalteromonas haloplanktis *is a psychrotolerant, marine, Gram-negative bacterium belonging to the γ-3-subgroup of proteobacteria. Currently it is one of the best investigated, cultivable representatives of the marine bacterioplankton and it can be considered to be one of the model organisms of cold-adapted bacteria [1].

Strain *P. haloplanktis *TAC125 (*Ph*TAC125) was isolated from a coastal seawater sample from the Antartic Ocean [2,3]. *Ph*TAC125 has been proposed as very valuable for biotechnical use due to its ability to grow fast at low temperatures. *P. haloplanktis *has a doubling time of 31 min at 20°C and is able to grow well at temperatures as low as 0°C [4].

In the recent years *Ph*TAC125 has been developed and established as a new alternative expression host [5]. A number of heterologous proteins could be produced in their soluble and active state in this organism, which were not or only poorly produced in *E. coli*, e.g. a mesophilic toluene o-xylene monooxygenase [6], human nerve growth factor (hNGF) [7] and Antarctic flavohemoglobin [8]. The construction of a shuttle vector system [3] and the identification of suitable constitutive promoters formed the basis to establish *P. haloplankti*s as cold-active expression system for recombinant protein production [9]. Recently a new two-component regulatory system based on L-malate was adapted for *Ph*TAC125 for the expression of heterologous proteins [10,11]. The fact that the cold-adapted *P. haloplanktis *proteome is enriched in asparagine residues compared with counterparts that grow at higher temperature, suggests the use of this psychrophilic organism for foreign protein production when deamidation should be at a minimum [4,12].

Recently it has been shown that the application of the C-terminus of the α-amylase from *P. haloplanktis *TAB23 enables a secretion of heterologous proteins into the extracellular medium by the Gram-negative host *Ph*TAC125 [13]. Moreover, the recombinant secretion system was recently improved by a mutant strain with reduced extracellular proteolytic activity [11].

Despite the increasing interest in *Ph*TAC125 the volumetric product yields are still poor due to low cell densities. To our knowledge only maximum cell densities of 5 to 6 g l^-1 ^have been reached [7] and no fed-batch process has been established so far. Process development with this organism is a challenge, as it does not grow on D-glucose [4] or on related sugars (e.g. D-fructose or D-xylose) [14]. The growth on arabinose, glycerol and glutamic acid as sole carbon source is also not possible [7]. *Ph*TAC125 seems to be well adapted to growth on rich media [4], and in all media described for this bacterium amino acids were used as carbon and nitrogen source.

The aim of this study was to develop a fed-batch fermentation process by using a feeding solution with a complex amino acid source. The replacement of peptones or yeast extract by casamino acids enabled a uniform growth behaviour of *P. haloplanktis *cell cultures. The use of a fed-batch strategy allowed *Ph*TAC125 to grow to a final optical density (OD_540_) of 28 (11 g l^-1 ^cell dry weight). This was a twofold improvement compared to the highest cell density reached for *Ph*TAC125 and an approximately eightfold increase compared to the standard cultivation method in shake flasks.

## Results

### Batch cultivation

The development of a fed-batch process for *P. haloplanktis *was a challenge as this organism does not grow on D-glucose nor related sugars (e.g. D-fructose or D-xylose), but must be cultivated on complex medium [7,14]. Furthermore, the metabolism of this cold-adapted bacterium is not well known. Currently *P. haloplanktis Ph*TAC125 is mostly grown on modified DSMZ 79 medium [14], a minimal synthetic sea water medium, supplemented with the complex amino acid source soy peptone.

**Figure 1:**
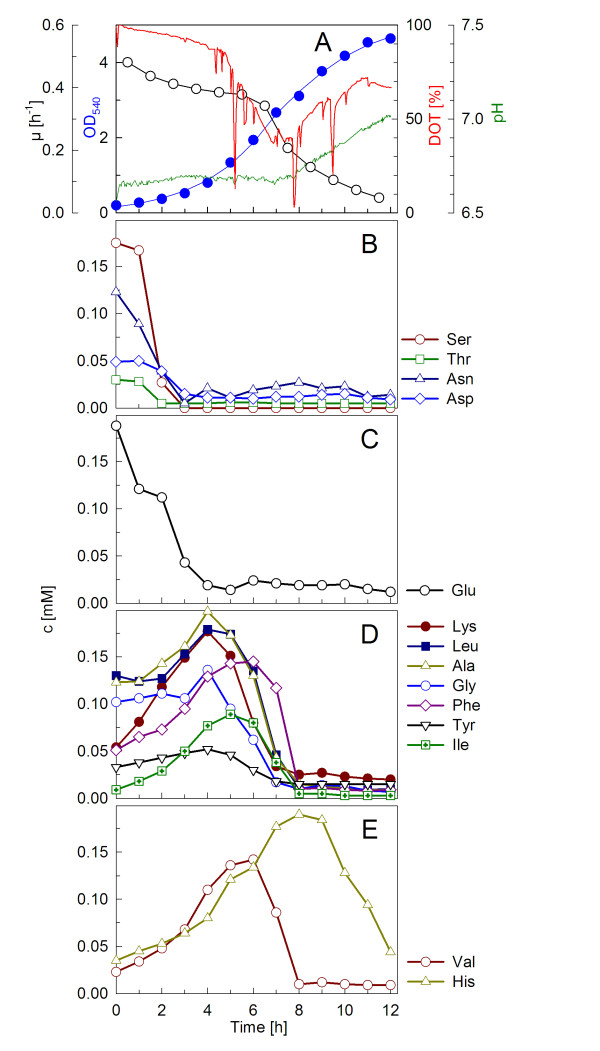
**Growth of *P. haloplanktis *strain TAC125 in a shake flask on modified DSMZ 79 medium supplemented with 5 g l^-1 ^soy peptone (SP)**. The cultivation was performed in a 3 l Erlenmeyer flask on a rotary shaker at 160 rpm at 16°C. DOT and pH were measured online with the SENBIT^®^-system. Spikes in the DOT curves derive from stops of the shaker during sampling. Composition and concentrations of the single amino acids of the soy peptone-containing medium (pepton N-Z-soy BL4/BL7) were analysed via GC-MS. (A) DOT, pH, growth curve and corresponding specific growth rate; (B-E) amino acids.

Figure 1 shows a typical shake flask batch-type cultivation of *Ph*TAC125 on standard DMSZ 79 medium with soy peptone. The growth curve was characterised by a number of metabolic switches which were indicated by clear changes in the pH and DOT values determined by the Senbit^® ^monitoring system [15].

As typical for complex medium-based batch cultures, exponential growth is only observed within the first 4 to 5 hours of the cultivation. The culture started to grow with a high specific growth rate of approximately 0.40 h^-1 ^for about three hours. During this phase a slight increase of pH was detected, possibly mainly due to the consumption of free glutamic acid (Figure 1A, C). The analysis of free amino acids in the soy peptone-containing culture medium further revealed that amino acids of the serine and the threonine pathway (threonine, asparagine, aspartic acid) decreased during this phase and stayed at a low and constant concentration thereafter (Figure 1B). In contrast the other amino acids accumulated in the growth medium during this phase (Figure 1D, E). Not the exhaustion of the serine, threonine, asparagine and aspartic acid but the limitation of glutamic acid seemed to induce the metabolic switch after about 4 hours. After this time the other amino acids were consumed with a higher rate, indicated by a decrease of their concentrations in the growth medium (Figure 1D, E). Interestingly, the consumption of oxygen increased significantly, causing a sharp bend in the DOT curve between 4 and 5 hours (see Figure 1A). Despite these changes, the specific growth rate remained high; only after the medium became exhausted in free amino acids around 7 to 8 hours the growth rate commenced to decrease. This is possibly a function of the rate of proteolytic release of amino acids from the complex amino acid source. During this phase the oxygen consumption decreased and the pH showed a clear increase. Finally, the growth ceased at about 11-12 hours.

**Figure 2:**
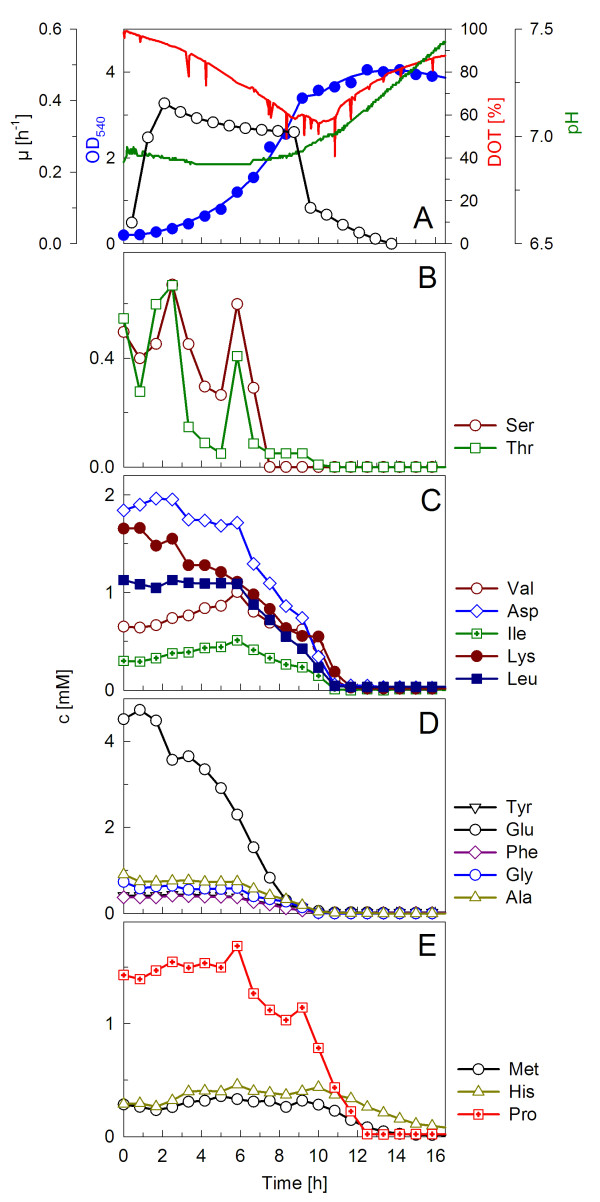
**Growth of *PhTAC125 *in a shake flask on modified DMSZ 79 medium supplemented with 5 g l^-1 ^CAA**. The cultivation was performed as described in the legend of Figure 1. The optical density, pH, DOT and dynamics of single amino acid concentrations are shown. (A) DOT, pH, growth curve and corresponding specific growth rate; (B-E) amino acids.

The metabolic consumption of single amino acids is difficult to determine in complex medium containing polypeptides since the actual concentration of free amino acids is a sum of peptide degradation on one side and microbial consumption on the other side. Therefore, soy peptone was exchanged with 5 g l^-1 ^casamino acids, which contain only free amino acids. A batch-culture with a similar final cell density (OD_540 _= 4) and growth rate (0.36 h^-1^) as in the soy peptone cultivations was obtained (cf. Figure 2). Although the casamino acids substrate had a different concentration of single amino acids compared to soy peptone, the early limiting and low concentrated amino acids remained the same. Serine and threonine were already exhausted after 7.5 h (Figure 2B). The levels of asparagine and tryptophane were below 0.1 M throughout the cultivation (data not shown). Glutamic acid appeared to be the most important amino acid controlling the growth. It was consumed with the highest rate compared to all other analysed amino acids (Figure 2D). When it was exhausted after about 9 hours the growth declined immediately. Between 9 and 14 hours, when the bulk of amino acids were exhausted, the OD_540 _increase was only marginal although respiration was still quite high.

During the first 10 h hours of cultivation a total amount of 1.7 g of pure amino acids (excluding methionine, histidine, asparagine and tryptophan, which were not consumed) were metabolised for the production of 1.28 g cell dry weight (CDW). With 0.66 g, glutamic acid contributed a proportion of approximately 39% to the CDW.

As a consequence of this experiment we concluded that a controlled growth of *P. haloplanktis *is possible when using a casamino acid-based medium. The use of casamino acids as a feed substrate is advantageous when compared to peptones due to the direct availability of the amino acids and the higher solubility (casamino acids, 80 to 100 g l^-1^; soy peptone, 50 g l^-1^). The growth on casamino acids-containing DMSZ medium showed a longer exponential growth phase, all single amino acids were consumed within a narrow time frame. Also, in contrast to the peptone medium, foam formation was negligible during the growth with casamino acids.

Evaluation of higher concentrations of casamino acids on higher biomass formation was subsequently determined. Doubling of the casamino acid concentration from 5 g l^-1 ^to 10 g l^-1 ^resulted in a doubling of the biomass concentration. A final optical density (OD_540_) of 8 was reached (data not shown). Hence casamino acids can be used as substrate for establishing a fed-batch process.

**Figure 3:**
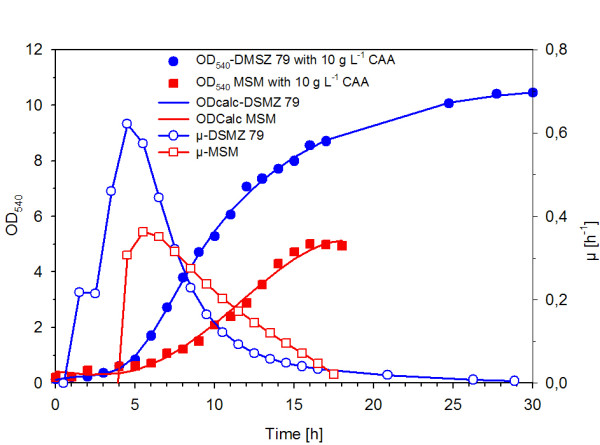
**Growth curves of *PhTAC125 *and corresponding specific growth rates of two different basic salt media each supplemented with 10 g l^-1 ^casamino acids**. The development of the optical density (■) and of the specific growth rate (□) of a FB mineral salt medium [16] in comparison to the optical density (●) and the specific growth rate (○) of the DSMZ 79 medium is shown. Both cultures were started with an initial optical density (OD_540_) of 0.2 and were incubated at 16°C and 200 rpm.

The so far described batch experiments with *P. haloplanktis *were performed on DMSZ 79 medium which is characterised by a very low phosphate concentration. For a fed-batch process a medium needs to be properly balanced to fulfil the growth needs. Media for high cell density cultivation have been traditionally buffered with phosphates, but so far such media have not been tested with *P. haloplanktis*. Therefore we investigated the growth on an *E. coli *fed-batch mineral salt medium (FB-MSM) [16] which was additionally complemented with 10 g l^-1 ^casamino acids (FB-CA). The phosphate concentration of this FB-MSM was 100-fold higher compared to the phosphate content of the DMSZ 79 medium, with 3.52 g l^-1 ^and 0.035 g l^-1 ^respectively. The growth on the fed-batch medium was possible but, in comparison to the growth on DSMZ 79 medium, the maximum specific growth rate was somewhat reduced (μ_max _= 0.35 h^-1 ^on DMSZ 79 medium with casamino acids; μ_max _= 0.28 h^-1 ^on the FB medium). The final cell yield was also 1.6-fold lower (OD_540 _of 5 compared to 8 on the DMSZ 79 medium) (cf. Figure 3). In further experiments FB-MSM was used for the fed-batch development.

### Fed-batch cultivation

**Figure 4:**
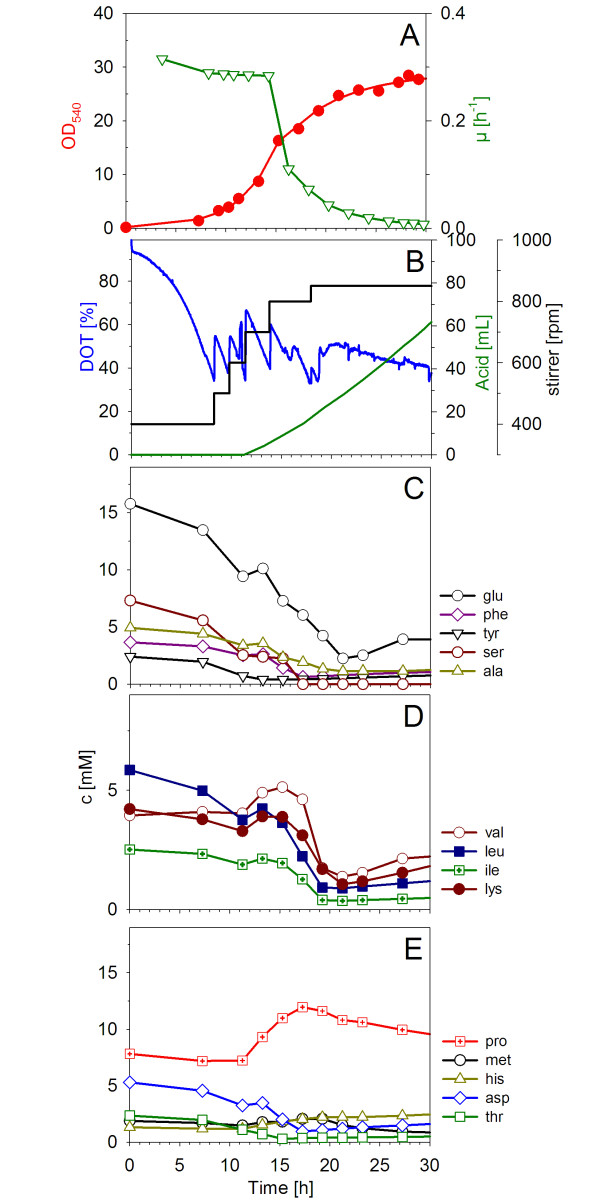
**Fed-batch fermentation of *Ph*TAC125 with a constant feeding of a highly-concentrated casamino acid solution (100 g l^-1^)**. The fed-batch cultivation was performed in the Applikon fermentation system with an initial operation volume of 1.4 l and an initial stirring rate of 400 rpm at 16°C. The feeding was started after 11.02 h with a constant rate of 0.028 l h^-1^. (A-B) development of growth parameters during FB-growth in the bioreactor; (A) growth curve and corresponding specific growth rate; (B) experimental data for DOT, acid and stirrer; (C-E) amino acids.

Casamino acids were used as a single feed, as the growth was purely dependent on the amino acid supply. To obtain the highest possible cell density, the casamino acid feed solution was concentrated as highly as possible, i.e. 80 to 100 g L^-1^. In Figure 4a fed-batch fermentation process with a constant feed rate of 0.028 L h^-1 ^(2 g L^-1 ^h^-1 ^casamino acids) is shown. Casamino acid feed with a constant rate was started at an OD_540 _of 5 after the increase of the DOT signal at 11 h, indicating the end of the batch phase.

At the start of constant feeding the addition of acid for the pH control was initiated (Figure 4B). The first hours of the fed-batch indicated a slight overfeeding as the concentrations of valine, leucine, isoleucine, lysine and proline increased during this period (Figure 4D, E). From 15 h and 21 h after inoculation the fed-batch process trended strongly towards a typical growth limitation profile. After 4 h from the start of feeding the growth rate slowed and the amino acids were consumed without further accumulation. The specific growth rate (μ) decreased from 0.2 h^-1 ^to 0.04 h^-1 ^indicative of substrate limitation. After 28.25 h the specific growth rate (μ) reached zero and the growth stopped (Figure 4A). A final optical density (OD_540_) of 28 was reached.

Because the final cell density in a nutrient-limited fed-batch process is always determined by the feed rate, further feed profiles with different feed rates were tested. In comparison to the used constant feed rate even an exponential feeding strategy did not significantly increase the cell density (Table 1). The next question was, whether the limit in cell density was caused by the casamino acid solution with a concentration of 100 g l^-1^. To answer this question another fed-batch experiment with casamino acid powder addition via single pulses of 10 g l^-1 ^(one pulse per hour) over a four-hour period was performed. The cell density was significantly increased to an optical density of 55 (data not shown). From this we conclude that the concentrated casamino acids in the feed solution limited the final cell density.

**Table 1:** Summary data of the fed-batch cultivations of *P. haloplanktis *TAC125 with different feeding profiles.

	Fed batch process	Length of batch period [h]	μ_max _in batch [h^-1^]	OD_540 _at feed start	Length of fed-batch phase [h]	Average μ during fed-batch [h^-1^]	Maximum OD_540_
1	constant feed	11.0	0.30	5	12	0.14	26
2	constant feed	13.1	0.29	5	9	0.2	27
3	constant feed	14.1	0.29	8	11	0.13	28
4	exponential feed	7.8	0.28	3	9	0.27	28

## Discussion

This study provides the first fed-batch fermentation strategy for *P. haloplanktis *TAC125 on complex medium. It is difficult to determine amino acid consumption rates from a medium which contains polymeric substances which are broken down into oligomers or monomers, thus we determined whether the peptone can be replaced by casamino acids. It could be demonstrated that casamino acids are a suitable substrate for a high cell density cultivation of *P. haloplanktis *cells. The exponential growth phase became more typical for a process which is controlled by one factor (i.e. by pH and DOT curves). No metabolic switches could be observed which were typical for the growth on peptone. Furthermore, the use of casamino acids was beneficial as they have a higher solubility than soy peptone (100 g l^-1 ^vs. 60 g l^-1^) and is more suitable for the development of a fed-batch process.

This is important, as the final cell density in a substrate-limited fed-batch is defined by the concentration of the nutrient in the feed solution. Aside from the substrate, the amount of inorganic ions must be increased to reach high cell densities. For this reason media must contain high salt concentrations in order to reach high cell densities. For *Ph*TAC125 we tested the usability of a fed-batch medium which has been used for cultivation of *E. coli*. Compared to the standard modified DSMZ 79 medium the bacterium showed a lower growth rate and a 50% reduction in the final cell yield, i.e. growth on the high salt medium was characterised by a higher maintenance requirement. It could be speculated that the higher maintenance requirement is due to the high salt content in this medium and thus a higher osmotic pressure. *Ph*TAC125 cells grew well in minimal medium over a large range of NaCl concentrations from 0% to 11%, but during the growth on rich media the growth of *Ph*TAC125 depended on the presence of salt with an optimal concentration between 1.5 and 3.5% [4]. Hence the salt concentration may affect the growth and could be a matter of further optimisation.

On the basis of the mineral salt-based fed-batch medium with casamino acids as a substrate, fed-batch cultivations were performed with a simple constant rate supply of a highly concentrated casamino acids solution (100 g l^-1^). Thus a final optical density of 28 was reached, corresponding to a cell dry weight of about 11 g l^-1^. This was a twofold increase of the CDW compared to the standard batch procedure on DMSZ 79 medium supplemented with soy peptone [14]. Preliminary kinetic simulations based on the yield coefficients for casamino acids determined from batch experiments indicated that this cell density is limited by the concentration of the casamino acids (data not shown). This was confirmed by a fed-batch experiment with casamino acids powder addition via single pulses of 10 g l^-1 ^over a four-hour period at the end of the cultivation. Thus, the cell density was significantly increased to an optical density of 55. If another substrate with a higher solubility would be found, the substrate concentration of the feeding solution could be increased. This would also considerably increase the cell density.

According to the suggestions for an optimisation of the medium towards a lower maintenance requirement and the search for a better soluble and a more distinct substrate it was important to determine the limiting components. With the help of the amino acid analysis some potential limiting amino acids could be identified, which seemed to satisfy the basic needs of *Ph*TAC125's carbon and energy supply. Of particular importance was glutamic acid which served in this study as the major carbon source. The yield coefficient Y_X/S _(g g^-1^) for glutamic acid was about 0.5, i.e. 0.66 mg l^-1 ^glutamic acid was consumed for the production of 1.28 g CDW. In comparison to the sum of the other single amino acids of the casamino acids-containing batch medium, glutamic acid contributed the biggest proportion of approximately 39% to biomass formation (see supplementary material).

The importance of glutamic acid was also noted from the fed-batch cultivations. Even when other amino acids accumulated (e.g. period from 11 h to 16 h in Figure 4) glutamic acid did not. Glutamic acid was the most strongly metabolised amino acid in all growth experiments. Furthermore, in early log phase of the cultures, serine, threonine, and aspartic acid were the first amino acids consumed and their limitation did not influence the growth significantly. Even the late limiting amino acids histidine, proline and methionine, which were indeed consumed after glutamic acid exhaustion, also did not affect the growth. During most cultivation experiments asparagine, tryptophan and methionine where negligible with concentrations lower than < 0.1 mM. The importance of glutamic acid is underpinned by the fact, that it belongs to one of the most frequently used amino acids for protein synthesis as determined by the proteome analysis of this psychrophilic γ-proteobacterium. On the other hand, the amino acids tryptophan, histidine and methionine belong to the most infrequently used ones [4] and as such, their absence did not affect the growth of *Ph*TAC125. Finally, the analysed data suggest that glutamic acid is an important carbon and nitrogen source for *P. haloplanktis*. Due to its high solubility of 740 g l^-1 ^sodium glutamate would be a perfect nutrient source in future fed-batch cultivations. Thus it would be interesting to see whether a fed-batch medium could be designed on the basis of sodium glutamate.

## Conclusion

The growth of *P. haloplanktis *to higher cell densities on complex medium is possible. A fed-batch fermentation strategy on an optimized medium with a final optical density of 28 could be established. The substrate concentration of the feeding solution was found to influence the maximal biomass yield considerably. The bottleneck for growing *P. haloplanktis *to high cell densities still remains the availability of a highly concentrated substrate with reduced substrate complexity. The amino acid analysis revealed glutamic acid, serine, threonine, aspartic acid and if applicable leucine as potential limiting amino acids. But only glutamic acid turned out to be the preferred amino acid with the highest influence on growth and thus is the most promising candidate as a highly concentrated carbon and nitrogen source for a fed-batch feeding solution. Finally, in combination with an optimised medium to lower the maintenance requirement, our results provide a good basis for further improvement of fed-batch processes based on this cold-adapted bacterium for lab-scale or industrial purposes.

## Methods

### Strains

The strain used in this study was *Pseudoalteromonas haloplanktis *TAC125 and was originally isolated from seawater of the Antarctic Ocean near the Dumont d'Urville station (66°40' S, 40°01' E) during an expedition by the institute Polaire Française [4]. This strain was kindly allocated from Georges Feller from the University of Liege (Belgium). The stock solution of the strain was stored in 20% [v/v] glycerol solution at -80°C.

### Media and cultivation conditions

Two precultures were used before main cultivations. The first preculture was grown over night (20 h) in 20 ml of ZoBell-medium in 100 ml Erlenmeyer flasks. For the second preculture the first preculture was 1:10, 1:100, 1:1000 and 1:10.000 diluted in modified DSMZ 79 medium supplemented with 5 g l^-1 ^soy peptone N-Z-soy BL4/7 (Sigma, Munich, Germany) with a final volume of 100 ml. After overnight cultivation the optical density (OD_540_) of the second preculture was determined and an exponentially growing culture (OD_540 _between 0.5 and 1.8) was chosen for the inoculation of the shake flasks or of the bioreactor. Both precultures were cultivated at 16°C in a rotary shaker with a rate of 200 rpm. For the cultivation the ZoBell medium [17] with the following composition was used: 750 ml artificial sea water [18] composed of 24 g l^-1 ^NaCl, 5.3 g l^-1 ^MgCl_2 _× 6H_2_O, 13.3 g l^-1 ^MgSO_4 _× 6H_2_O, 0.7 g l^-1 ^KCl, 0.02 g FeSO_4 _× 7H_2_O and 5 g l^-1 ^peptone N-Z-Soy BL4/BL7, 1 g l^-1 ^yeast extract, 0.01 g FePO_4_. Before sterilisation the pH was adjusted to 7.6. The medium based on Schatz [19], which was also described as DMSZ 79 medium [14] consisted of 5 g l^-1 ^peptone N-Z-Soy BL4/BL7, 0.14 g l^-1 ^KH_2_PO_4_^, ^1 g l^-1 ^NH_4_NO_3_^, ^10 g l^-1 ^NaCl, 0.2 g l^-1 ^MgSO_4 _× 7H_2_O, 0.01 g l^-1 ^FeSO_4 _× 7H_2_O and 0.01 g l^-1 ^CaCl_2 _× 2H_2_O. Before sterilisation the pH was adjusted to 7.0. Foam formation was a problem during all cultivation experiments, thus the silicone anti-foam emulsion from SERVA was used as an antifoam agent.

The mineral salt fed-batch medium [16] was prepared in distilled water containing (in g l^-1^): K_2_HPO_4 _14.6, NaH_2_PO_4 _× 2 H_2_O 3.6, Na_2_SO_4 _× 10 H_2_O 4.54, (NH_4_)_2_SO_4 _2.47, NH_4_Cl 0.5, (NH_4_)_2_-H-citrate 1.0 and an initial casamino acid concentration of 10 g l^-1^. The pH was adjusted to pH 7.0. After heat sterilisation 2 ml l^-1^of 1 M MgSO_4_, 5 ml l^-1 ^of 0.2 M KH_2_PO_4 _and 2 ml l^-1 ^trace element solution were added through a sterile filter (0.2 μm). The trace element solution was also prepared with distilled water containing in g l^-1^: CaCl_2 _× 2 H_2_O 0.5, ZnSO_4 _× 7 H_2_O 0.18, MnSO_4 _× H_2_O 0.1, Na-EDTA 20.1, FeCl_2 _× 6 H_2_O 16.7, CuSO_4 _0.1 and CoCl_2 _× 6 H_2_O 0.15.

### Bioreactor fed-batch cultures

The cultivations were mainly performed in the Applikon fermentation system (Applikon, Schiedam, The Netherlands) in a glass bioreactor with a total volume of 3 litres and a maximal operating volume of 2 to 2.5 litres. The bioprocess was controlled via the Applikon ADI 1030 biocontroller. The stirred tank reactor was equipped with 3 standard six-blade Rushton turbines and four baffles. In addition the bioreactor was equipped with the standard pH-, pO_2_-, level- and temperature sensors for monitoring of the bioprocess. The cultivation temperature was always 16°C and the pH was kept at 7.0 mainly by one-sided feed of 1 M sulfuric acid. The dissolved oxygen tension (DOT) was kept above 20% during the fed-batch fermentation. Foam formation was prevented by distinct addition of small amounts of antifoam agent when needed. The specific supply of the cells with substrate was performed by a specific feeding of high-concentrated substrate solution. The consumed volumes of the substrate feeding solution, of the sulfuric acid and of the antifoam solution were monitored via a combination of the weight difference of the single corresponding supply flasks before and after the fermentation and the calibration parameters of the corresponding feed pumps.

Fed-batch process were started with an initial volume of 1.4 l of defined mineral salt fed-batch fermentation medium. After sterilisation and addition of the trace elements and MgSO_4 _the bioreactor was inoculated with the second preculture to an initial OD_540 _of 0.1 to 0.2. The feed solution contained 80 to 100 g l^-1 ^casamino acids (ROTH GmbH+Co.KG) and 100 ml l^-1 ^of 10× concentrated salt solution of the mineral salts of the above described mineral salt medium. During all fed-batch cultivations additional 2 ml l^-1 ^of 1 M MgSO_4_-solution were added approximately for every 10 units increase of OD_540 _and additional 5 ml l^-1 ^of 3.8 M NH_4_SO_4_-solution were added approximately for every 20 units increase of OD_540_. The addition of these salts should avoid a potential limitation of these salts during the fed-batch cultivation.

The feeding of the substrate solution with dissolved casamino acids was mainly performed via a constant addition of the feed solution. In detail 4 constant feeding profiles and one exponential feeding profile were used. The feeding profiles were adapted to a typical growth curve of *Ph*TAC125 to avoid overfeeding or to control the growth by nutrient limitation. All constant feeding profiles were started with an initial feed rate of 2 g l^-1^h^-1 ^and were changed during the beginning of the stationary phase to (a) 4, (b) 1.7, or (c) 2.9 g l^-1^h^-1^. The exponential feeding was started with an initial ratio of 0.07 g l^-1 ^h^-1 ^and was then stepwise increased in an exponential curve shape for every 2 hours until the maximal feed rate of 3.5 g l^-1^h^-1 ^was attained. The beginning of the constant feed phase depended on the duration of the batch-phase and was launched after the limitation signal (increase of DOT signal). The exponential feeding profile was started 4 h after inoculation.

### Sampling

During the exponential growth phase of the batch phase samples for the determination of the optical density (OD_540_) were taken hourly. During the feeding phase samples for the OD_540 _were only taken every 3 to 5 hours. Additionally and in parallel samples for cell dry weight determination, for microscopy and for amino acid and metabolite analysis were taken regularly. Sterile sampling was performed via a sterile port of the bioreactor to which a sterile syringe was connected.

### Analysis of cell growth

Cell growth was monitored spectrophotometrically by measurement of the optical density at 540 nm (OD_540_) and via the cell dry weight. One unit of OD_540 _corresponds to a cell dry weight of 0.36 g l^-1^. The specific growth rate μ was calculated from the fitted OD_540 _values.

The cell dry weight was determined gravimetrically from 2 ml samples after washing with 0.9% NaCl, centrifugation and drying of the cell pellets at 80°C for 24 h.

### Analysis of extracellular amino acids and metabolite concentration

The samples for the amino acid and metabolite analysis were centrifuged at maximal rpm. The supernatant was filtered with a sterile filter (0.2 μm) and afterwards stored at -20°C until further analysis.

Levels of free amino acids were measured in extracellular samples using the phenomenex^® ^EZ:faast kit (EZ:faast GC/MS free (physiological) amino acid kit) by gas chromatography/mass spectrometry. Quantification was done with MetaQuant 1.2 and norvaline was used as internal standard. Methodical details are described by Liebeke et al. [20].

Additionally, selected samples were subjected to ^1^H-NMR measurements for extracellular metabolite profiling, they were collected as mentioned above. Preparation and analysis of filtered samples was done as described previously [20]. Briefly, after addition of 200 μl phosphate-buffer (pH 7.0) to 400 μl sample in a glass tube (Norell Standard Series, length 7 inch) an acquisition of ^1^H-NMR spectra was performed. Metabolite quantification was done by comparing the areas of designated signals to the internal standard trimethylsilylpropionic acid d_4_, included in the added phosphate-buffer with a concentration of 1.0 mM.

## Competing interests

The authors declare that they have no competing interests.

## Authors' contributions

BW carried out most of the experiments and was mainly responsible for the preparation of the manuscript. AH participated crucially in the fed-batch fermentation experiments. MLa and MLi carried out the GC-MS and NMR-based metabolite analyses.

PN conducted the set-up of the fed-batch experiments. He was strongly involved in the data analyses and the preparation of the manuscript. TS initiated and co-conducted this study and was involved in the writing of the manuscript. All authors have read and approved the final manuscript.
